# Botfly myiasis after travel to Bolivia

**DOI:** 10.1002/ski2.253

**Published:** 2023-06-28

**Authors:** Aheli Chattopadhyay, Jason F. Wang, Maria L. Wei

**Affiliations:** ^1^ Department of Dermatology University of California San Francisco San Francisco California USA; ^2^ Dermatology Service San Francisco VA Health Care System San Francisco California USA

## Abstract

Following a trip to Bolivia, a 32‐year‐old woman developed a left lower leg ulcer with a sensation of movement within the lesion. After being seen by four primary care providers, she was referred to dermatology 7 weeks after her return from Bolivia. At that time, she was found to have a 5 mm weeping ulcer, with a live larva visible at the base. We conducted a punch biopsy for botfly removal, after which the patient healed well. Herein we discuss the ways in which clinical presentation, history of travel, dermoscopy, and ultrasound can contribute to diagnosing botfly myiasis. While treatment of botfly infestation is not required, we discuss the importance of shared decision‐making in considering treatment, as well as methods for extraction, including mechanical or surgical removal, which may help to reduce patient anxiety and the risk for secondary infection. As global travel resumes to levels prior to the Covid‐19 pandemic, it is important for dermatologists to be aware of the presenting symptoms and treatment of tropical skin disorders.

## Graphical abstract

A 32‐year‐old woman developed a left lower leg ulcer with a sensation of movement within the lesion after travel to Bolivia, which was diagnosed as botfly infestation and removed via punch biopsy. We use this case as a guide to discuss how clinical presentation, history of travel, dermoscopy, and ultrasound can contribute to diagnosing botfly myiasis, and the various options for safe removal that are available for those who choose to pursue treatment. With global travel increasing, it is important for dermatologists to recognise signs and symptoms of tropical skin disorders.

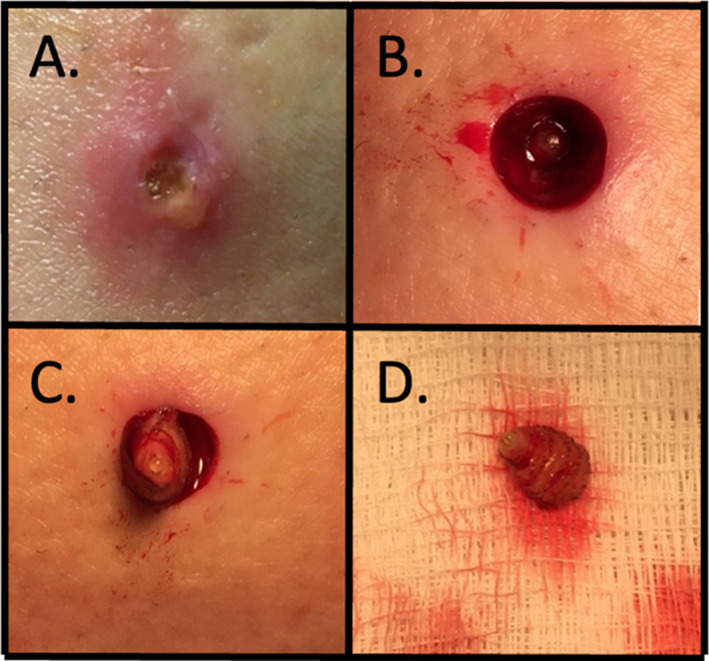

## 1 CASE REPORT

**FIGURE 1:**
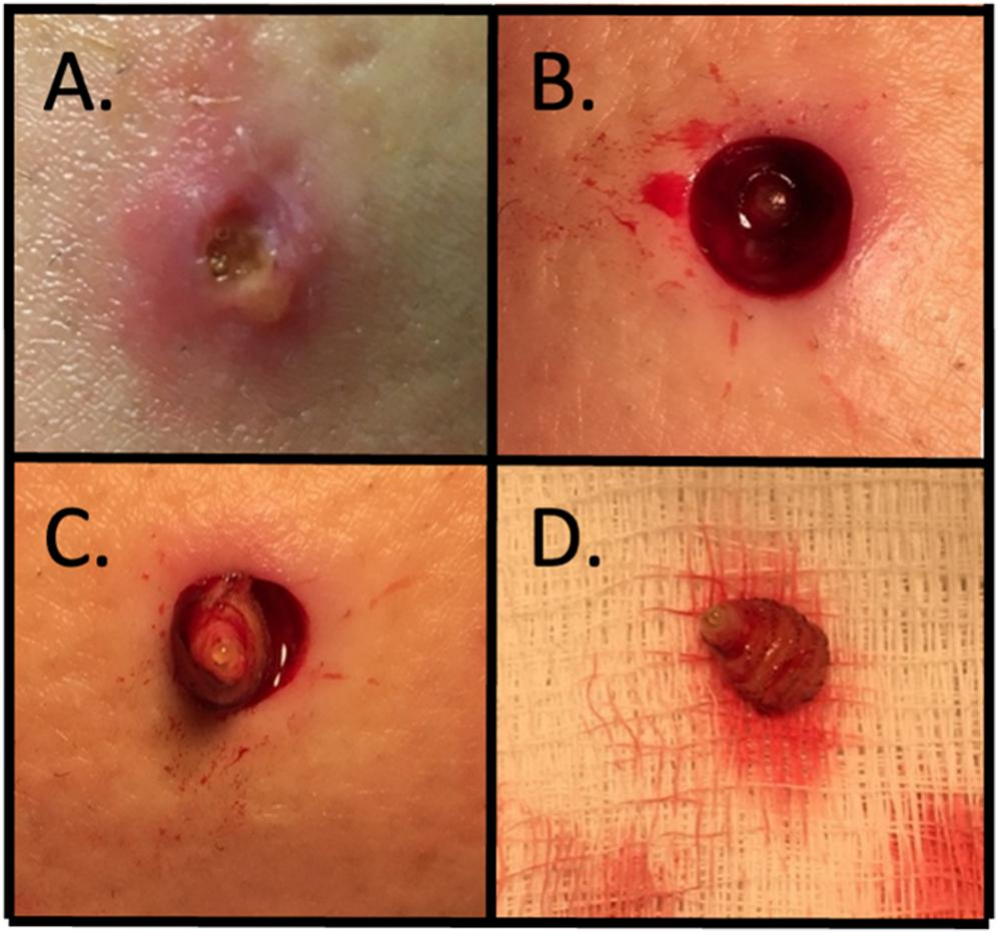
Initial presentation and extraction of botfly larva. (a) Clinical presentation: visible tip of larva at bottom of ulcer. (b and c) Punch biopsy and extraction. (d) Extracted botfly larva.

A 32‐year‐old woman travelled to Bolivia, including a visit to the jungle. Three weeks after returning, she was evaluated by a primary care provider for a concern of a left lower leg lesion, due to a sensation of movement within the lesion. Bacterial wound culture was negative, and Neosporin and 2% mupirocin ointment were prescribed without improvement. The patient was subsequently seen by three other primary care providers, without a diagnosis. She was then referred to dermatology 7 weeks after returning from Bolivia, at which time a 5 mm weeping ulcer was evident, revealing a live larva (Figure 1a). The lesion was not associated with pruritis or pain at that time. A punch biopsy (Figure 1b, c) facilitated the extraction of the larva (Figure 1d). Pathology confirmed myiasis. The patient returned to clinic for follow‐up 11 days later for suture removal, and the biopsy site was healing well; she remains well 6 years later.

## 2 DISCUSSION

This case was typical of *Dermatobia hominis* botfly myiasis in several ways: (1) recent travel to Bolivia, as botfly are endemic to Central and South America and Africa, with a majority of cases reported from Belize; (2) presence of a slow‐growing papule with central opening producing serosanguinous fluid with pressure; (3) sensation of movement within the lesion, (4) lack of fever or lymphadenopathy, and (5) lack of response to antibiotic treatment. The lower body has been implicated in about 30% of studied cases.^1^ Beyond clinical presentation and history of travel, dermoscopy may show a central opening, dilated blood vessels, and yellow structures with barb‐like spines, suggestive of botfly larvae.^2^ Ultrasound showing a subcutaneous “wriggling” worm can be utilised to confirm the diagnosis and number of parasites and guide removal.^1^, ^3^ Most cases of botfly involve skin; 15% affect additional organs including lung, gastrointestinal, and visual systems,^4^ not seen in this patient. Differential diagnoses for botfly infestations of the skin in the literature include furuncle, epidermal cyst, foreign body, pyoderma, infected insect bite, cat scratch disease, cutaneous leishmaniasis, or other parasitic infection, including tungiasis.^1^ Education regarding clinical signs is important to prevent diagnostic delay.

Botfly myiasis can resolve without treatment, as botflies feed on host tissue for five to 10 weeks, then eject from host tissue. There is room for shared decision‐making in determining treatment, and patient anxiety is an important consideration. Methods for extraction can involve mechanical or surgical removal,^1^ including punch biopsy, forceps extraction, and manual expression. We counselled our patient that intervention was not necessary; however, she had tremendous unease and expressed great relief when offered extraction by biopsy. Patients sometimes pursue at‐home larvae removal, which increases risk of incomplete removal and secondary infection. Appropriately diagnosing botfly infestation and offering optional removal in clinic can ease patients' anxiety, provide the opportunity for relevant counselling, and potentially minimise the risk for secondary infection.

## AUTHOR CONTRIBUTIONS

**Aheli Chattopadhyay**: Conceptualization (equal); Formal analysis (lead); Investigation (lead); Methodology (equal); Visualization (equal); Writing – original draft (lead); Writing – review & editing (equal). **Jason F. Wang**: Conceptualization (equal); Methodology (equal); Writing – review & editing (equal). **Maria Wei**: Conceptualization (equal); Methodology (equal); Resources (lead); Software (lead); Supervision (lead); Visualization (equal); Writing – review & editing (equal).

## CONFLICT OF INTEREST STATEMENT

None to declare.

## ETHICS STATEMENT

Not applicable.

## Data Availability

Data sharing is not applicable to this article as no new data were created or analyzed in this study.
